# Model of THz Magnetization Dynamics

**DOI:** 10.1038/srep22767

**Published:** 2016-03-09

**Authors:** Lars Bocklage

**Affiliations:** 1 Deutsches Elektronen-Synchrotron DESY, Notkestraβe 85, 22607 Hamburg, Germany; 2 The Hamburg Centre for Ultrafast Imaging, Luruper Chaussee 149, 22761 Hamburg, Germany

## Abstract

Magnetization dynamics can be coherently controlled by THz laser excitation, which can be applied in ultrafast magnetization control and switching. Here, transient magnetization dynamics are calculated for excitation with THz magnetic field pulses. We use the ansatz of Smit and Beljers, to formulate dynamic properties of the magnetization via partial derivatives of the samples free energy density, and extend it to solve the Landau-Lifshitz-equation to obtain the THz transients of the magnetization. The model is used to determine the magnetization response to ultrafast multi- and single-cycle THz pulses. Control of the magnetization trajectory by utilizing the THz pulse shape and polarization is demonstrated.

Interactions of light and matter play a crucial role in the control of ultrafast solid state processes. With the advent of ultra short laser pulses a new time scale of magnetization dynamics became accessible^1^,^2^,^3^,^4^. The interaction of laser pulses induces magnetization dynamics either by heating of the electronic system or by non-thermal photo- or optomagnetic effects^5^. One possible coupling of optical laser pulses to the magnetization is given by the inverse Faraday effect in ferrimagnetic garnets^3^. However, optical laser pulses often induce thermal effects in metals and for non-canted antiferromagnets the inverse Faraday effect is not applicable. For these materials, a direct Zeeman coupling of the magnetic field of the photon to the magnetization is achieved in the low THz regime^6^,^7^ and no thermal processes have been observed in these experiments. The resulting transient magnetization dynamics became accessible with the ability to generate high-power THz coherent laser pulses from optical lasers^8^,^9^ or electron sources^10^,^11^ whose pulse width is tunable down to single cycle pulses^10^,^12^,^13^,^14^. The frequency spectrum of these pulses is in a range where ferromagnetic eigenmodes, the ferromagnetic resonance or spin waves, are not effectively excited. Thus, magnetization dynamics follows on the time scale of the field pulse stimulus that is orders of magnitude faster than ferromagnetic resonances^7^. For antiferromagnetic materials^6^,^15^ resonant excitation is achieved in the THz regime because of the high resonance frequencies of antiferromagnetic magnons. In both cases transient magnetic states are excited by THz pulses.

These ultra fast time-scales require a theoretical approach that handles transient magnetization dynamics at these time scales. The Landau-Lifshitz-Gilbert (LLG) equation^16^,^17^ describes magnetization dynamics in the micromagnetic model and covers typical length and time scales of today’s technological important magnetic materials. It was shown that ultrafast magnetization dynamics are described by the LLG as long as the interactions are non-thermal and arise from a time-variation of the effective field^6^,^7^,^18^. This holds for THz laser pulses^6^,^7^.

Based on the LLG we develop an analytical model that covers transient magnetic states which couple directly to magnetic field stimuli. We solve the LLG in spherical coordinates and the effective magnetic field is expressed via the derivatives of the free energy density^19^,^20^ similar to the formulation of the ferromagnetic resonance by Smit and Beljers^21^. In this way we derive a general solution for transient magnetic states that are independent of actual sample properties and of the explicit knowledge of the internal fields. Magnetization dynamics can be directly calculated for any sample geometry, field configuration, or anisotropy directly from the free energy density. The THz pulse shape and polarization provide a full vectorial control of the magnetization on the sub-picosecond time scale.

## Theory

**Figure 1:**
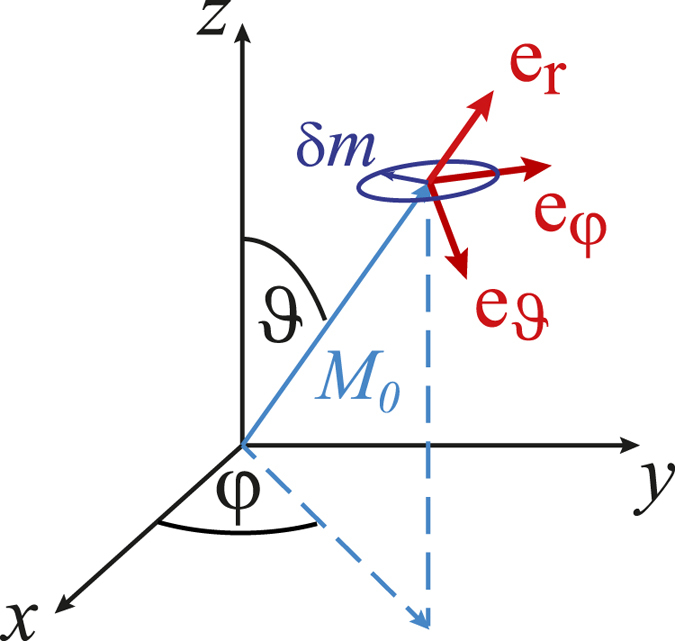
Spherical coordinate system with polar angle *ϑ* and azimuthal angle *φ*. The basis vectors 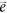
_*r,ϑ,φ*_ (red) are fixed by the static magnetization 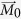 (light blue). They indicate the directions of the dynamic magnetization 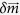 (dark blue) and the dynamic field 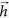, which are both in the 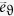
−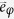-plane.

Magnetization dynamics are described for small changes of the magnetization vector 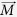. Therefore, the magnetization is split in a constant part 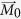 and a small temporal changing component 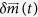. In spherical coordinates the polar and azimuthal angles of the magnetization are *ϑ*(*t*) = *ϑ*_0_ + *δϑ*(*t*) and *φ*(*t*) = *φ*_0_ + *δφ*(*t*), respectively. The angles of the equilibrium magnetization are *ϑ*_0_ and *φ*_0_. The coordinate system is shown in Fig. 1.

The Landau-Lifshitz equation in spherical coordinates is^22^,^23^


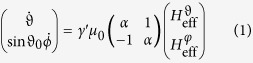


with the Gilbert damping parameter^17^ *α* (Spin transport phenomena, like spin pumping, can alter the effective damping). The radial component has been omitted as it is zero. The vacuum permeability is *μ*_0_, the gyromagnetic ratio is *γ* with *γ*′ = *γ*/(1 + *α*^2^), and the effective field is 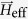. The dynamic magnetization components are calculated from the dynamic angles via 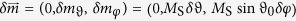.

Here, we solve the differential equation and calculate the response of a uniform magnetization to a temporally varying and spatially homogeneous small external field 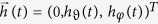. The effective field is given by the derivative of the free energy density *F* and by 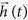 as 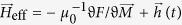. The exchange field is not taken into account, i.e., antiferromagnets and ferrimagnets as well as spin waves are not included in the model. Calculations on THz dynamics of antiferromagnets can be found in ref. 24. The individual components of the effective field are 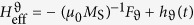 and 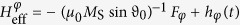. The indices at *F* indicate partial derivatives around equilibrium positions. Without exchange and with the approximation of the free energy density from the ansatz of Smit and Beljers^21^,^25^, the system of differential equations becomes linear and can be written as


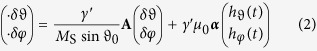


with


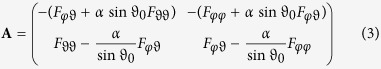


and


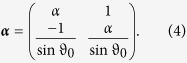


The solution for the free transient states of the magnetization is calculated to


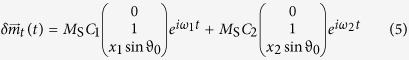


where constants *C*_1,2_ have to satisfy the given initial condition. The eigenfrequencies are


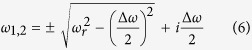


with the Smit-Beljers resonance frequency^25^


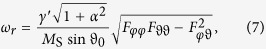


as well as the resonance linewidth due to Gilbert damping^25^,^26^


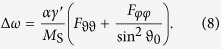


The factors *x*_1,2_ are


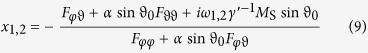


and give the ratio of the dynamic angular components *δφ*/*δϑ* = *x*_1,2_ of the resonant modes *ω*_1,2_.

The dynamic susceptibility ***χ*** of ferromagnets^22^,^27^ is small for frequencies off-resonance and, thus, resonant dynamics dominate for broadband pulsed excitation. For stimuli with THz pulses however, the Fourier amplitudes at ferromagnetic resonances (FMR or spin waves) are vanishingly small. Hence, the prominent dynamics do not longer occur on a time scale of the resonance frequency but on that of the coherent THz laser pulse and a much faster coherent magnetization control can be obtained.

To calculate the response of the magnetization to THz pulses from Eq. (2), the dynamic magnetic field is approximated as a product of a harmonic signal and a Gaussian^8^,^9^,^10^,^11^,^28^
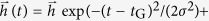

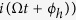
, characterized by the width *σ* and the temporal shift *t*_G_ of the envelope as well as the frequency Ω and the phase *ϕ*_*h*_ of the harmonic. The solution of the dynamic magnetization gets


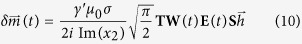


with the matrices


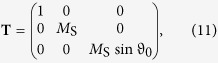



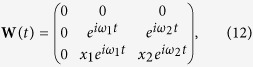



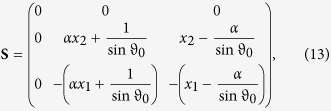


and


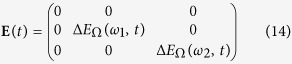


where Δ*E*_Ω_(*ω*, *t*) = *E*_Ω_(*ω*, *t*) − *E*_Ω_(*ω*, 0) with


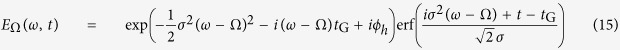


and with the error function erf(*z*) with complex argument *z*. For Ω = 0 and *ϕ*_*h*_ = 0 one arrives at the solution for a pure Gaussian pulse excitation.

## Results

**Figure 2:**
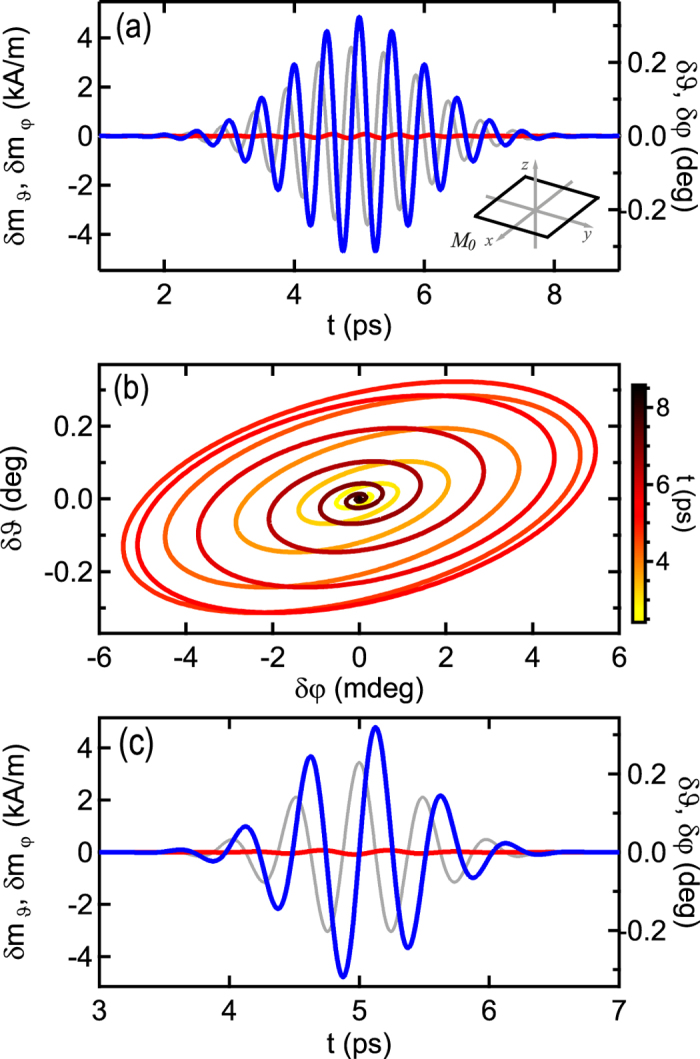
Magnetization dynamics for an excitation with a linearly polarized THz pulse with *σ* = 1 ps, *t*_G_ = 5 ps, Ω = 2 THz, and *ϕ*_*h*_ = − Ω*t*_G_ + *π*/2. Blue and red indicate polar *ϑ* and azimuthal *φ* components, respectively. The grey line depicts the temporal evolution of the excitation field with a field amplitude of 400 mT, where the ordinate for the field ranges from −600 to 600 mT. The inset shows the orientation of the film and of the magnetization in the coordinate system. (**b**) Magnetization trajectory in the phase space of the dynamic angles. The magnetization performs a few cycles and returns to rest. (**c**) Magnetization dynamics for an excitation with a THz pulse with the same parameters as in (**a**) except that *σ* = 500 fs and *φ*_*h*_ = −Ω*t*_G_.

The response to a THz magnetic field pulse with a frequency of Ω = 2 THz and a width of *σ* = 1 ps is shown in Fig. 2 for a thin film system, one of the most widely used systems. For the computation the values of a polycrystalline permalloy film are used that is oriented in the *xy*-plane. Parallel to the static magnetization 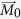 along the *x*-axis an uniaxial in-plane anisotropy *K*_*u*_ and an external field *μ*_0_*H*_ext_ = 10 mT are set. The partial derivatives of the free energy density are then given by *F*_*φφ*_ = *μ*_0_*M*_S_(*H*_ext_ + *H*_*A*_), *F*_*ϑϑ*_ = *μ*_0_*M*_S_(*H*_ext_ + *M*_S_ + *H*_*A*_), and *F*_*φϑ*_ = 0 with *H*_*A*_ = 2*K*_*u*_/(*μ*_0_*M*_S_). Typical material parameters are *M*_S_ = 860 kA/m, *K*_*u*_ = 400 J/m^3^, *α* = 0.007 that yield a resonance frequency *ω*_*r*_/2*π* = 3.06 GHz, much smaller than the THz frequency. *δm*_*φ*_ is the in-plane and *δm*_*ϑ*_ the out-of-plane component. The dynamic field (0, *h*_*φ*_) points in-plane perpendicular to the static magnetization, i.e., the THz pulse is linearly polarized. Its amplitude *μ*_0_*h*_*φ*_ is set to 400 mT. Such high field amplitudes are achievable with intense laser radiation^7^ and necessary to induce magnetization changes in the order of a percent because the susceptibility ***χ*** is typically small in the THz regime. Therefore, magnetization dynamics are small and our linear assumption is well justified even at these high field amplitudes. As the model is linear, the magnetization amplitude scales linearly with the field amplitude. The response to a shorter pulse with another phase is shown in Fig. 2(c). Here, the relative phase of both magnetization components to the excitation field stays the same, so THz pulses coherently drive the magnetization.

As observed in Fig. 2 the out-of-plane oscillation is dominant, which is in contrast to the larger in-plane oscillation that one obtains at resonant excitation^29^. The amplitude of the in-plane dynamics is smaller than the amplitude of the out-of-plane dynamics by a factor of about 50. This behavior is due to the reaction of the magnetization to a magnetic field. The instantaneous direction of motion points out of the plane spanned by 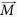 and 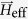, i.e. the out-of-plane direction in the given example. The THz field alters so fast that the motion can be considered as instantaneous in the timescale of the pulse and the dominant dynamics are out-of-plane. Further, the magnetization follows the stimulus in phase. The calculated response corresponds to experimental findings by Vicario *et al.*^7^. Figure 2(b) shows the oscillation path in phase space. The magnetization performs a few cycles before returning to equilibrium and is at rest after the multi-cycle pulse is completed.

**Figure 3:**
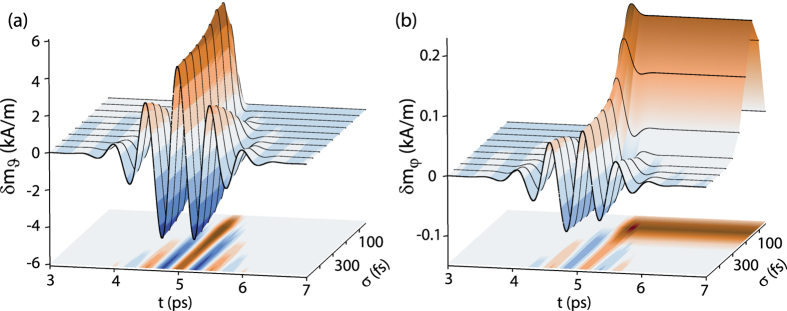
Magnetization dynamics for an excitation with a field from a THz pulse with *σ* ranging from 50 fs to 500 fs in steps of 50 fs. The other parameters are as before. Time-dependent dynamic magnetization of the (**a**) polar *ϑ* and (**b**) azimuthal *φ* component.

**Figure 4:**
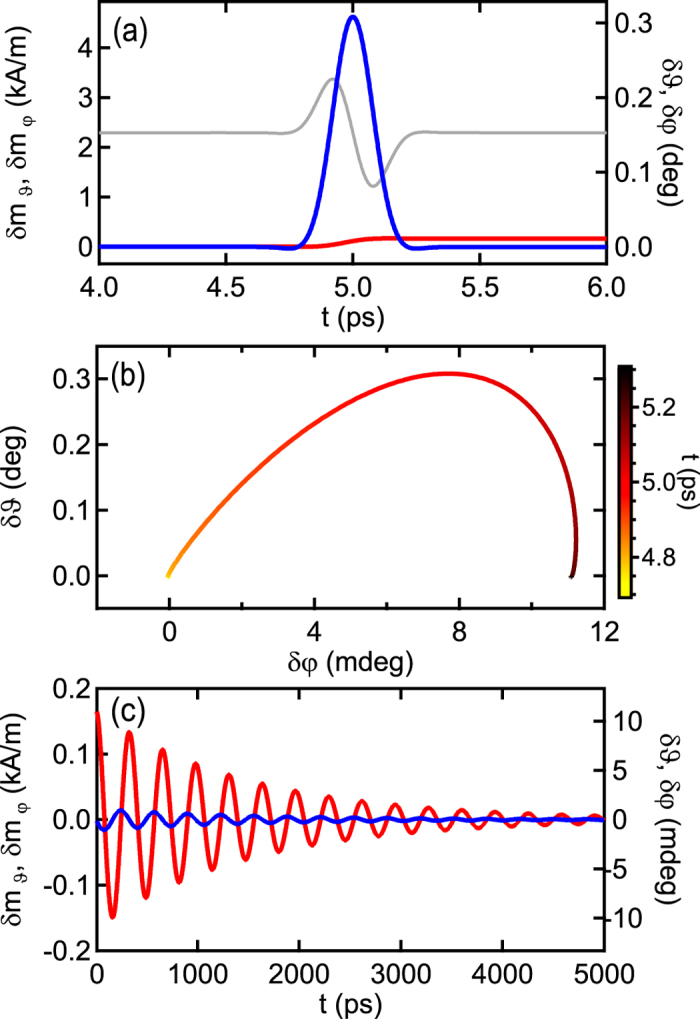
Magnetization dynamics for excitation with a THz pulse with *σ* = 100 fs. Other parameters are as before. Time-dependent magnetization are shown on a short time scale of the laser pulse in (**a,b**) and on the time scale of the ferromagnetic resonance in (**c**).

For a field pulse approaching the single-cycle limit the situation differs. The evolution of the magnetization for various *σ* is shown in Fig. 3. Short excitation pulses are finished before the magnetization returns to equilibrium and the in-plane magnetization has an offset. From Fig. 3 it is observed that the magnetization cannot return to rest for pulses whose *σ* is smaller than the THz period. Consequently, THz pulses that are short compared to the period 2*π*/Ω lead to a ringing of the magnetization. Which magnetization component is offset depends on the phase *φ*_*h*_. Here, it is set to *φ*_*h*_ = −Ω*t*_G_ + *π*/2 to satisfy ∫^∞^_−∞_*B*_THz_(*t*)d*t* = 0 by *B*_THz_(*t*) being antisymmetric about *t*_G_. Figure 4(a,b) depict the magnetization for a pulse with *σ* = 100 fs on the short time scale of the pulse. This is comparable to pulses that have been experimentally achieved^30^. From the phase space plot it is visible that the magnetization passes only about a quarter cycle after the pulse is over. From this moment the transient states, described by Eq. (5), dominate the dynamics. The out-of-plane magnetization resonantly oscillates into equilibrium after the pulse as observed from Fig. 4(c) which shows the dynamics on the time scale of the resonance frequency. For decreasing width, the pulse approaches a delta function like stimulus and a broadband excitation is obtained whose Fourier component at *ω*_*r*_ increases. For fast pump-probe experiments or technical applications the ringing of the magnetization can be avoided by setting a proper pulse width with respect to the harmonic.

To extend the degree of freedom in THz magnetization control the light’s polarization is utilized in the following. A vectorial control of the magnetization by THz light was demonstrated for resonant excitation of antiferromagnetic magnons^15^. Here, it is shown that THz light can also be used to control the magnetization trajectory for non-resonant stimuli of ferromagnets by utilizing the THz pulse polarization, which becomes more and more tunable^31^.

**Figure 5:**
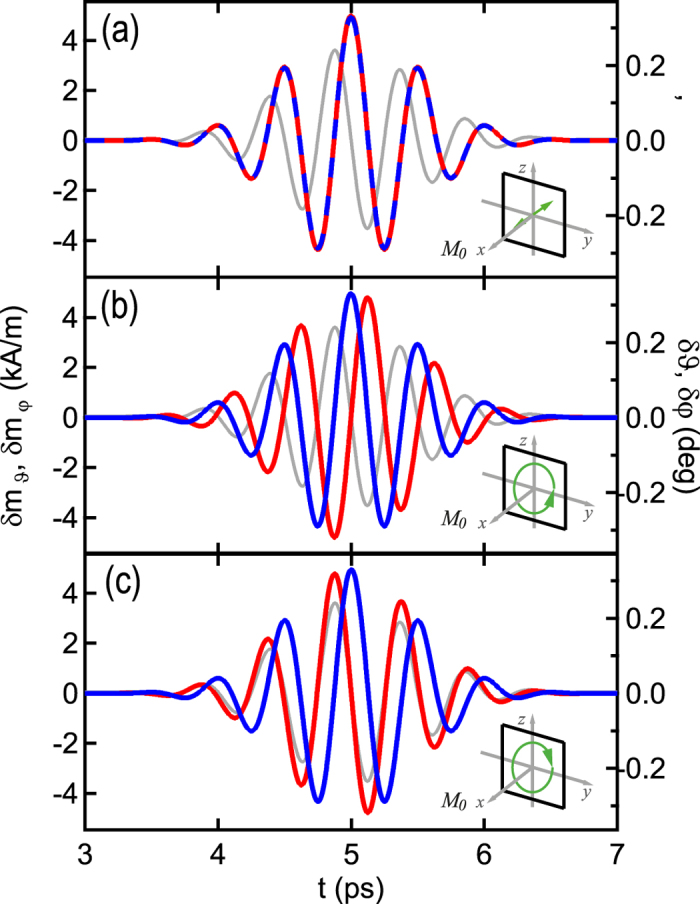
Magnetization dynamics for excitation with linearly (a) and circularly (b,c) polarized THz pulses with *σ* = 500 fs impinging perpendicularly to the film plane. The phase *β*_*ϑ*_ is set to zero in (**a**), to *π*/2 in (**b**), and to −*π*/2 in (**c**) and the amplitudes *μ*_0_*h*_*ϑ*_ and *μ*_0_*h*_*φ*_ are both 400 mT. The out-of-plane external field is 1.1 *M*_S_ to align the magnetization out-of-plane along the *x*-axis. Blue and red indicate polar *ϑ* and azimuthal *φ* components (along the *z* and *y*-directions), respectively. Note that 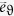 points in negative *z*-direction. Here, the grey line depicts the *h*_*φ*_ component. The oscillation direction of the THz field in the *y*-*z*-plane is indicated by green arrows in the insets. The magnetization oscillation is perpendicular to the THz field in (**a**). The magnetization circulation direction in (**b**,**c**) equals that of the THz field.

An elliptical polarization is introduced via 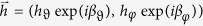. Here, we consider a circular polarization and choose 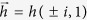, which preserves the phase of *h*_*φ*_ being the exciting field in the linearly polarized example. Using circular polarized light impinging perpendicularly to the film surface will not change the results shown previously because the additional dynamic in-plane component is aligned parallel to the static in-plane magnetization. It will not initiate dynamics. Therefore, the static magnetization is aligned out-of-plane by an external magnetic field (which could also be achieved by an intrinsic perpendicular anisotropy of the film). This configuration results in the magnetization dynamics shown in Fig. 5 for three different polarizations (For the calculation, the film is oriented in the *y*-*z*-plane). Now both magnetization components equally react to the pulse. Only the THz pulse polarization has to be altered to obtain either linear or circular precession modes, whereby each mode can be addressed individually. *m*_*φ*_ is altered by the phase shifted dynamic field *h*_*ϑ*_ and again the response of the magnetization is determined by the dynamic field component perpendicular to it. Most interestingly this enables a vectorial control of the magnetization by utilizing the polarization. The analytical calculation given here is only valid for the Gaussian THz pulse with one defined polarization state. However, experimentally it has been shown that THz pulses can be pulse-shaped to a large extent^31^ and more complicated magnetization trajectories become accessible. The off-resonant THz excitation should facilitate the highly tunable manipulation of the magnetization, as there is no unique and dominant mode in the THz regime, thus, enabling to arbitrarily shape the magnetization trajectory.

In principle the solution is not restricted to a special parameter range and gives the response to a directly coupled dynamic magnetic field as long as the reactions of the magnetic system are not too large, i.e., the linear model is valid. This holds up to approximately 20° ref. 29 which lies well in the experimentally obtainable range. Care must be taken to which extent other thermal or non-thermal effects contribute to magnetization dynamics in laser-induced experiments above the low THz regime. A review on these effects can be found in ref. 5.

## Summary

An analytical model for transient magnetization dynamics of a homogeneously magnetized sample based on the Landau-Lifshitz equation with Gilbert damping is derived. The solution to the equation of motion is formulated via the partial derivatives of the free energy density and is given for multi- and single-cycle THz laser pulses. It represents a general solution to the excitation profile independent on the actual sample properties. Therefore, the trajectory can be calculated for any material, shape, or magnetocrystalline anisotropy of the sample as soon as its energy terms are known. Measured magneto-optical signals can be compared to the formalism which can help to disentangle magnetization dynamics initiated by a laser pulse that couples either directly or indirectly to the magnetization. The control of the magnetization trajectory by THz pulses is demonstrated. This can be achieved via the temporal shape of the pulse as well as its polarization. THz pulses are essential to future applications of ultrafast magnetization control and switching.

## Additional Information

**How to cite this article**: Bocklage, L. Model of THz Magnetization Dynamics. *Sci. Rep.* **6**, 22767; doi: 10.1038/srep22767 (2016).
